# Supplementary material for: Specific Phylotypes of Saprolegnia parasitica Associated with Atlantic Salmon Freshwater Aquaculture
Source: J Fungi (Basel). 2024 Jan 9;10(1):57. doi: 10.3390/jof10010057 (PMC10820671; doi:10.3390/jof10010057)
Supplement: Supplementary file 1 [file jof-10-00057-s001.zip › jof-2682412-supplementary.pdf]

Table S1. 412 isolates from Scottish Atlantic salmon aquaculture sites. Metadata includes the aquaculture site the isolate was from, the date of sampling, isolate source (skin, fin or tank water), isolate species, epizootic status during time of sampling and the ITS accession number. The sample phylotype is also given for all *S. parasitica* samples. Species were identified via NCBI BLAST and then confirmed by alignment with reference samples from Sandoval-Sierra et al., 2014b.

Isolate I.D.	Sampling Site	Date Isolated	Isolate Source	Isolate Species	*S. parasitica* Phylotype	Epizootic Status	ITS Accession #
AA.01.09	A	20/11/2017	Skin	*Saprolegnia parasitica*	*S5*	N/a	OQ678408
AA.02.04	A	23/11/2017	Fin	*Mortierella hyalina*		N/a	OQ678409
AA.02.16	A	23/11/2017	Fin	*Mortierella hyalina*		N/a	OQ678410
AA.03.16	A	18/12/2017	Fin	*Mortierella hyalina*		N/a	OQ678411
AA.04.02	A	23/01/2018	Fin	*Mortierella hyalina*		N/a	OQ678412
AA.04.02.12	A	23/01/2018	Tank water	*Mortierella hyalina*		N/a	OQ678413
AA.04.10	A	23/01/2018	Fin	*Mortierella sp.5*		N/a	OQ678414
AA.04.18	A	23/01/2018	Fin	*Mortierella sp.11*		N/a	OQ678415
AA.32.02.08	A	10/07/2018	Tank water	*Pythium coloratum*		N/a	OQ678416
AA.35.02.02	A	07/08/2018	Tank water	*Trichoderma viride*		N/a	OQ678417
AA.35.02.04	A	07/08/2018	Tank water	*Saprolegnia parasitica*	*S3*	N/a	OQ678418
AA.39.02	A	03/09/2018	Fin	*Mortierella elongata*		N/a	OQ678419
AA.39.06	A	03/09/2018	Fin	*Mortierella elongata*		N/a	OQ678420
AA.39.16	A	03/09/2018	Fin	*Mortierella elongata*		N/a	OQ678421
AA.39.18	A	03/09/2018	Fin	*Mortierella elongata*		N/a	OQ678422
AA.43.02.11	A	01/10/2018	Tank water	*Mortierella hyalina*		N/a	OQ678423
AA.48.16	A	02/11/2018	Fin	*Mortierella sossaunensis*		N/a	OQ678424
AA.52.02.20	A	28/11/2018	Tank water	*Pythium sp.5*		N/a	OQ678425

AA.52.08	A	28/11/2018	Fin	*Pythium sp.5*		N/a	OQ678426
AA.52.12	A	28/11/2018	Fin	*Saprolegnia parasitica*	*S2*	N/a	OQ678427
AA.52.28	A	28/11/2018	Fin	*Pythium sp.5*		N/a	OQ678428
AA.62.02.12	A	05/02/2019	Tank water	*Saprolegnia delica*		Enzootic	OQ678429
AA.67.10	A	19/03/2019	Fin	*Mortierella hyalina*		Enzootic	OQ678430
AA.67.15	A	19/03/2019	Skin	*Mortierella hyalina*		Enzootic	OQ678431
AA.71.02	A	15/04/2019	Fin	*Achlya sp.3*		Enzootic	OQ678432
AA.83.02.24	A	12/07/2019	Tank water	*Pythium coloratum*		Enzootic	OQ678433
AA.94.02	A	04/10/2019	Fin	*Pythium sp.5*		Enzootic	OQ678434
AA.94.06	A	04/10/2019	Fin	*Saprolegnia diclina*		Enzootic	OQ678435
AA.94.08	A	04/10/2019	Fin	*Pythium sp.5*		Enzootic	OQ678436
AA.94.10	A	04/10/2019	Fin	*Pythium sp.5*		Enzootic	OQ678437
AA.94.11	A	04/10/2019	Skin	*Pythium sp.5*		Enzootic	OQ678438
AA.94.12	A	04/10/2019	Fin	*Pythium sp.5*		Enzootic	OQ678439
AA.94.18	A	04/10/2019	Fin	*Pythium anandrum*		Enzootic	OQ678440
AA.98.06	A	30/10/2019	Fin	*Pythium sp.5*		Enzootic	OQ678441
AA.98.08	A	30/10/2019	Fin	*Pythium sp.5*		Enzootic	OQ678442
AA.98.10	A	30/10/2019	Fin	*Pythium sp.5*		Enzootic	OQ678443
AA.103.02	A	27/11/2019	Fin	*Pythium undulatum*		Enzootic	OQ678444
AA.103.04	A	27/11/2019	Fin	*Pythium sp.5*		Enzootic	OQ678445
AA.103.10	A	27/11/2019	Fin	*Saprolegnia diclina*		Enzootic	OQ678446
AA.103.12	A	27/11/2019	Fin	*Saprolegnia diclina*		Enzootic	OQ678447
AA.103.18	A	27/11/2019	Fin	*Pythium sp.5*		Enzootic	OQ678448
BB.01.02.06	B	29/09/2017	Tank water	*Pythium sp.5*		Enzootic	OQ678449
BB.01.02.07	B	29/09/2017	Tank water	*Pythium sp.5*		Enzootic	OQ678450

BB.01.02.09	B	29/09/2017	Tank water	*Pythium torulosum*		Enzootic	OQ678451
BB.01.07	B	29/09/2017	Skin	*Mortierella sp.7*		Enzootic	OQ678452
BB.01.17	B	29/09/2017	Skin	*Saprolegnia parasitica*	*S2*	Enzootic	OQ678453
BB.11.02	B	06/12/2017	Fin	*Saprolegnia parasitica*	*S2*	Enzootic	OQ678454
BB.11.08	B	06/12/2017	Fin	*Saprolegnia parasitica*	*S2*	Enzootic	OQ678455
BB.13.02.12	B	22/12/2017	Tank water	*Saprolegnia diclina*		Enzootic	OQ678456
BB.18.02.10	B	24/01/2018	Tank water	*Saprolegnia diclina*		Enzootic	OQ678457
BB.26.02.07	B	22/03/2018	Tank water	*Pythium coloratum*		Enzootic	OQ678458
BB.26.02.15	B	22/03/2018	Tank water	*Mortierella hyalina*		Enzootic	OQ678459
BB.28.02.02	B	04/04/2018	Tank water	*Pythium sp.5*		Enzootic	OQ678460
BB.36.04	B	25/05/2018	Fin	*Saprolegnia parasitica*	*S2*	Epizootic	OQ678461
BB.36.08	B	25/05/2018	Fin	*Saprolegnia parasitica*	*S2*	Epizootic	OQ678462
BB.36.14	B	25/05/2018	Fin	*Saprolegnia parasitica*	*S2*	Epizootic	OQ678463
BB.38.02.06	B	13/06/2018	Tank water	*Pythium coloratum*		Enzootic	OQ678464
BB.38.02.24	B	13/06/2018	Tank water	*Pythium coloratum*		Enzootic	OQ678465
BB.42.02.02	B	11/07/2018	Tank water	*Pythium coloratum*		Epizootic	OQ678466
BB.42.02.17	B	11/07/2018	Tank water	*Pythium coloratum*		Epizootic	OQ678467
BB.50.10	B	05/09/2018	Fin	*Pythium coloratum*		Enzootic	OQ678468
BB.55.02.24	B	09/10/2018	Tank water	*Pythium undulatum*		Enzootic	OQ678469
BB.63.06	B	04/12/2018	Fin	*Mortierella turficola*		Enzootic	OQ678470
BB.63.07	B	04/12/2018	Skin	*Pythium sp.4*		Enzootic	OQ678471
BB.63.16	B	04/12/2018	Fin	*Saprolegnia parasitica*	*S2*	Enzootic	OQ678472
BB.66.02.19	B	03/01/2019	Tank water	*Pythium coloratum*		Enzootic	OQ678473
BB.75.10	B	01/03/2019	Fin	*Saprolegnia parasitica*	*S2*	Enzootic	OQ678474
BB.75.18	B	01/03/2019	Fin	*Saprolegnia parasitica*	*S2*	Enzootic	OQ678475

BB.79.02	B	28/03/2019	Fin	*Saprolegnia parasitica*	*S2*	Enzootic	OQ678476
BB.79.06	B	28/03/2019	Fin	*Saprolegnia parasitica*	*S2*	Enzootic	OQ678477
BB.79.14	B	28/03/2019	Fin	*Saprolegnia parasitica*	*S2*	Enzootic	OQ678478
BB.79.18	B	28/03/2019	Fin	*Saprolegnia parasitica*	*S2*	Enzootic	OQ678479
BB.95.02.18	B	18/07/2019	Tank water	*Pythium coloratum*		Enzootic	OQ678480
BB.99.02.03	B	14/08/2019	Tank water	*Pythium coloratum*		Enzootic	OQ678481
CC.01.02.20	C	12/01/2018	Tank water	*Mortierella hyalina*		Enzootic	OQ678482
CC.01.10	C	12/01/2018	Fin	*Saprolegnia parasitica*	*S2*	Enzootic	OQ678483
CC.01.18	C	12/01/2018	Fin	*Saprolegnia parasitica*	*S2*	Enzootic	OQ678484
CC.02.02	C	19/01/2018	Fin	*Saprolegnia parasitica*	*S2*	Enzootic	OQ678485
CC.02.04	C	19/01/2018	Fin	*Saprolegnia parasitica*	*S2*	Enzootic	OQ678486
CC.02.10	C	19/01/2018	Fin	*Saprolegnia parasitica*	*S2*	Enzootic	OQ678487
CC.02.14	C	19/01/2018	Fin	*Saprolegnia parasitica*	*S2*	Enzootic	OQ678488
CC.02.16	C	19/01/2018	Fin	*Saprolegnia parasitica*	*S2*	Enzootic	OQ678489
CC.03.01	C	05/02/2018	Skin	*Saprolegnia parasitica*	*S2*	Epizootic	OQ678490
CC.03.15	C	05/02/2018	Skin	*Saprolegnia parasitica*	*S2*	Epizootic	OQ678491
CC.03.16	C	05/02/2018	Fin	*Saprolegnia parasitica*	*S2*	Epizootic	OQ678492
CC.03.17	C	05/02/2018	Skin	*Saprolegnia parasitica*	*S2*	Epizootic	OQ678493
CC.03.18	C	05/02/2018	Fin	*Saprolegnia parasitica*	*S2*	Epizootic	OQ678494
CC.04.06	C	20/02/2018	Fin	*Saprolegnia ferax*		Enzootic	OQ678495
CC.12.02	C	17/05/2018	Fin	*Pythium flevoense*		Enzootic	OQ678496
CC.16.04	C	22/06/2018	Fin	*Saprolegnia ferax*		Enzootic	OQ678497
CC.16.08	C	22/06/2018	Fin	*Pythium flevoense*		Enzootic	OQ678498
CC.16.17	C	22/06/2018	Skin	*Saprolegnia ferax*		Enzootic	OQ678499
CC.19.02	C	16/07/2018	Fin	*Pythium flevoense*		Enzootic	OQ678500

CC.21.02.21	C	N/a	Tank water	*Saprolegnia ferax*		Enzootic	OQ678501
CC.24.02	C	16/08/2018	Fin	*Pythium flevoense*		Enzootic	OQ678502
CC.24.02.03	C	16/08/2018	Tank water	*Saprolegnia ferax*		Enzootic	OQ678503
CC.24.02.13	C	16/08/2018	Tank water	*Pythium flevoense*		Enzootic	OQ678504
CC.24.02.17	C	16/08/2018	Tank water	*Saprolegnia ferax*		Enzootic	OQ678505
CC.24.02.18	C	16/08/2018	Tank water	*Saprolegnia ferax*		Enzootic	OQ678506
CC.24.02.21	C	16/08/2018	Tank water	*Saprolegnia ferax*		Enzootic	OQ678507
CC.24.02.22	C	16/08/2018	Tank water	*Pythium flevoense*		Enzootic	OQ678508
CC.24.06	C	16/08/2018	Fin	*Saprolegnia ferax*		Enzootic	OQ678509
CC.24.08	C	16/08/2018	Fin	*Saprolegnia ferax*		Enzootic	OQ678510
CC.24.10	C	16/08/2018	Fin	*Saprolegnia ferax*		Enzootic	OQ678511
CC.24.14	C	16/08/2018	Fin	*Pythium flevoense*		Enzootic	OQ678512
CC.24.16	C	16/08/2018	Fin	*Saprolegnia ferax*		Enzootic	OQ678513
CC.24.18	C	16/08/2018	Fin	*Saprolegnia ferax*		Enzootic	OQ678514
CC.28.02	C	12/09/2018	Fin	*Saprolegnia ferax*		Enzootic	OQ678515
CC.28.06	C	12/09/2018	Fin	*Saprolegnia parasitica*	*S6*	Enzootic	OQ678516
CC.28.12	C	12/09/2018	Fin	*Saprolegnia parasitica*	*S6*	Enzootic	OQ678517
CC.30.02.10	C	25/09/2018	Tank water	*Saprolegnia ferax*		Enzootic	OQ678518
CC.30.02.19	C	25/09/2018	Tank water	*Saprolegnia ferax*		Enzootic	OQ678519
CC.30.04	C	25/09/2018	Fin	*Saprolegnia parasitica*	*S2*	Enzootic	OQ678520
CC.30.06	C	25/09/2018	Fin	*Pythium flevoense*		Enzootic	OQ678521
CC.30.08	C	25/09/2018	Fin	*Saprolegnia parasitica*	*S2*	Enzootic	OQ678522
CC.30.10	C	25/09/2018	Fin	*Saprolegnia ferax*		Enzootic	OQ678523
CC.30.12	C	25/09/2018	Fin	*Pythium flevoense*		Enzootic	OQ678524
CC.30.14	C	25/09/2018	Fin	*Saprolegnia parasitica*	*S2*	Enzootic	OQ678525

CC.30.18	C	25/09/2018	Fin	*Pythium flevoense*		Enzootic	OQ678526
CC.31.09	C	27/09/2018	Skin	*Pythium flevoense*		Epizootic	OQ678527
CC.34.12	C	10/10/2018	Fin	*Pythium flevoense*		Epizootic	OQ678528
CC.34.16	C	10/10/2018	Fin	*Saprolegnia parasitica*	*S2*	Epizootic	OQ678529
CC.38.01	C	05/11/2018	Skin	*Trametes versicolor*		Epizootic	OQ678530
CC.38.12	C	05/11/2018	Fin	*Saprolegnia parasitica*	*S2*	Epizootic	OQ678531
CC.38.16	C	05/11/2018	Fin	*Saprolegnia parasitica*	*S2*	Epizootic	OQ678532
CC.41.02	C	29/11/2018	Fin	*Saprolegnia parasitica*	*S2*	Enzootic	OQ678533
CC.41.04	C	29/11/2018	Fin	*Saprolegnia parasitica*	*S2*	Enzootic	OQ678534
CC.41.06	C	29/11/2018	Fin	*Saprolegnia ferax*		Enzootic	OQ678535
CC.41.12	C	29/11/2018	Fin	*Saprolegnia ferax*		Enzootic	OQ678536
CC.41.14	C	29/11/2018	Fin	*Saprolegnia ferax*		Enzootic	OQ678537
CC.42.07	C	30/11/2018	Skin	*Mortierella hyalina*		Enzootic	OQ678538
CC.42.08	C	30/11/2018	Fin	*Mortierella hyalina*		Enzootic	OQ678539
CC.42.12	C	30/11/2018	Fin	*Mortierella hyalina*		Enzootic	OQ678540
CC.42.18	C	30/11/2018	Fin	*Saprolegnia parasitica*	*S2*	Enzootic	OQ678541
CC.43.06	C	06/12/2018	Fin	*Saprolegnia ferax*		Enzootic	OQ678542
CC.43.08	C	06/12/2018	Fin	*Saprolegnia parasitica*	*S2*	Enzootic	OQ678543
CC.43.10	C	06/12/2018	Fin	*Saprolegnia ferax*		Enzootic	OQ678544
CC.43.12	C	06/12/2018	Fin	*Saprolegnia ferax*		Enzootic	OQ678545
CC.43.16	C	06/12/2018	Fin	*Achlya sp.4*		Enzootic	OQ678546
CC.47.02.24	C	02/01/2019	Tank water	*Saprolegnia ferax*		Enzootic	OQ678547
CC.47.04	C	02/01/2019	Fin	*Saprolegnia ferax*		Enzootic	OQ678548
CC.47.18	C	02/01/2019	Fin	*Pythium flevoense*		Enzootic	OQ678549
CC.54.02	C	27/02/2019	Fin	*Saprolegnia parasitica*	*S2*	Enzootic	OQ678550

CC.54.06	C	27/02/2019	Fin	*Saprolegnia ferax*		Enzootic	OQ678551
CC.54.16	C	27/02/2019	Fin	*Saprolegnia parasitica*	*S2*	Enzootic	OQ678552
CC.63.06	C	29/07/2019	Fin	*Saprolegnia ferax*		Enzootic	OQ678553
CC.63.12	C	29/07/2019	Fin	*Saprolegnia ferax*		Enzootic	OQ678554
CC.63.14	C	29/07/2019	Fin	*Saprolegnia parasitica*	*S6*	Enzootic	OQ678555
CC.63.18	C	29/07/2019	Fin	*Saprolegnia parasitica*	*S6*	Enzootic	OQ678556
CC.65.18	C	07/08/2019	Fin	*Saprolegnia ferax*		Enzootic	OQ678557
CC.66.02.05	C	09/08/2019	Tank water	*Saprolegnia parasitica*	*S6*	Enzootic	OQ678558
CC.66.02.06	C	09/08/2019	Tank water	*Saprolegnia ferax*		Enzootic	OQ678559
CC.66.02.07	C	09/08/2019	Tank water	*Saprolegnia parasitica*	*S6*	Enzootic	OQ678560
CC.66.02.08	C	09/08/2019	Tank water	*Saprolegnia parasitica*	*S6*	Enzootic	OQ678561
CC.66.02.09	C	09/08/2019	Tank water	*Saprolegnia parasitica*	*S6*	Enzootic	OQ678562
CC.66.02.11	C	09/08/2019	Tank water	*Saprolegnia parasitica*	*S6*	Enzootic	OQ678563
CC.66.02.18	C	09/08/2019	Tank water	*Saprolegnia parasitica*	*S6*	Enzootic	OQ678564
CC.66.02.21	C	09/08/2019	Tank water	*Saprolegnia parasitica*	*S6*	Enzootic	OQ678565
CC.66.02.23	C	09/08/2019	Tank water	*Saprolegnia parasitica*	*S6*	Enzootic	OQ678566
CC.70.02	C	30/08/2019	Fin	*Saprolegnia parasitica*	*S6*	Enzootic	OQ678567
CC.70.08	C	30/08/2019	Fin	*Pythium flevoense*		Enzootic	OQ678568
CC.70.08	C	30/08/2019	Fin	*Pythuim flevoense*		Enzootic	OQ678569
CC.70.10	C	30/08/2019	Fin	*Saprolegnia parasitica*	*S6*	Enzootic	OQ678570
CC.70.11	C	30/08/2019	Skin	*Pythium flevoense*		Enzootic	OQ678571
CC.74.02	C	27/09/2019	Fin	*Saprolegnia parasitica*	*S6*	Enzootic	OQ678572
CC.74.02.24	C	27/09/2019	Tank water	*Hypholoma fasciculare*		Enzootic	OQ678573
CC.74.06	C	27/09/2019	Fin	*Saprolegnia parasitica*	*S6*	Enzootic	OQ678574
CC.74.12	C	27/09/2019	Fin	*Saprolegnia ferax*		Enzootic	OQ678575

CC.74.18	C	27/09/2019	Fin	*Saprolegnia parasitica*	*S6*	Enzootic	OQ678576
CC.78.04	C	23/10/2019	Fin	*Saprolegnia parasitica*	*S6*	Enzootic	OQ678577
CC.78.06	C	23/10/2019	Fin	*Saprolegnia ferax*		Enzootic	OQ678578
CC.78.08	C	23/10/2019	Fin	*Saprolegnia parasitica*	*S6*	Enzootic	OQ678579
CC.78.10	C	23/10/2019	Fin	*Saprolegnia parasitica*	*S6*	Enzootic	OQ678580
CC.78.16	C	23/10/2019	Fin	*Saprolegnia parasitica*	*S6*	Enzootic	OQ678581
CC.78.18	C	23/10/2019	Fin	*Saprolegnia parasitica*	*S6*	Enzootic	OQ678582
CC.85.16	C	11/12/2019	Fin	*Achlya sp.4*		Enzootic	OQ678583
CC.85.17	C	11/12/2019	Skin	*Saprolegnia parasitica*	*S6*	Enzootic	OQ678584
CC.87.02.11	C	07/01/2020	Tank water	*Saprolegnia parasitica*	*S6*	Enzootic	OQ678585
CC.87.02.22	C	07/01/2020	Tank water	*Saprolegnia parasitica*	*S6*	Enzootic	OQ678586
CC.87.16	C	07/01/2020	Fin	*Saprolegnia parasitica*	*S6*	Enzootic	OQ678587
CC.87.18	C	07/01/2020	Fin	*Saprolegnia parasitica*	*S6*	Enzootic	OQ678588
DD.02.02.05	D	10/11/2017	Tank water	*Pythium sp.5*		Enzootic	OQ678589
DD.05.02	D	12/12/2017	Fin	*Mortierella turficola*		Enzootic	OQ678590
DD.05.04	D	12/12/2017	Fin	*Mortierella turficola*		Enzootic	OQ678591
DD.05.08	D	12/12/2017	Fin	*Mortierella elongata*		Enzootic	OQ678592
DD.06.01	D	08/01/2018	Skin	*Cadophora luteo-olivacea*		Enzootic	OQ678593
DD.37.04	D	17/07/2018	Fin	*Saprolegnia parasitica*	*S2*	Epizootic	OQ678594
DD.39.18	D	24/07/2018	Fin	*Saprolegnia parasitica*	*S2*	Epizootic	OQ678595
DD.41.12	D	15/08/2018	Fin	*Saprolegnia parasitica*	*S2*	Enzootic	OQ678596
DD.44.18	D	07/09/2018	Fin	*Mortierella turficola*		Epizootic	OQ678597
DD.57.10	D	28/11/2018	Fin	*Saprolegnia parasitica*	*S4*	Enzootic	OQ678598
DD.71.10	D	26/02/2019	Fin	*Saprolegnia parasitica*	*S2*	Enzootic	OQ678599
DD.75.18	D	25/03/2019	Fin	*Saprolegnia parasitica*	*S4*	Enzootic	OQ678600

DD.85.03	D	24/07/2019	Skin	*Mortierella macrocystis*		Enzootic	OQ678601
DD.85.17	D	24/07/2019	Skin	*Mortierella sossaunensis*		Enzootic	OQ678602
DD.90.28	D	20/08/2019	Fin	*Pythium sp.6*		Enzootic	OQ678603
EE.01.02.11	E	23/11/2017	Tank water	*Pythium coloratum*		Enzootic	OQ678604
EE.01.02.20	E	23/11/2017	Tank water	*Achlya sp.3*		Enzootic	OQ678605
EE.01.02.21	E	23/11/2017	Tank water	*Saprolegnia diclina*		Enzootic	OQ678606
EE.03.02.16	E	01/02/2018	Tank water	*Saprolegnia diclina*		Enzootic	OQ678607
EE.03.02.24	E	01/02/2018	Tank water	*Saprolegnia diclina*		Enzootic	OQ678608
EE.04.02.08	E	06/02/2018	Tank water	*Saprolegnia diclina*		Enzootic	OQ678609
EE.08.02.09	E	06/03/2018	Tank water	*Achlya sp.3*		Enzootic	OQ678610
EE.15.06	E	31/05/2018	Fin	*Mortierella sp.5*		Enzootic	OQ678611
EE.19.06	E	12/07/2018	Fin	*Saprolegnia parasitica*	*S6*	Enzootic	OQ678612
EE.19.10	E	12/07/2018	Fin	*Achlya sp.3*		Enzootic	OQ678613
EE.21.16	E	09/08/2018	Fin	*Saprolegnia parasitica*	*S6*	Enzootic	OQ678614
FF.00.43	F	23/11/2017	Skin	*Pythium sp.2*		Enzootic	OQ678615
FF.01.12	F	23/11/2017	Fin	*Saprolegnia parasitica*	*S6*	Enzootic	OQ678616
FF.01.16	F	23/11/2017	Fin	*Saprolegnia parasitica*	*S6*	Enzootic	OQ678617
FF.04.02.02	F	31/05/2018	Tank water	*Saprolegnia parasitica*	*S6*	Enzootic	OQ678618
FF.05.02.04	F	18/06/2018	Tank water	*Saprolegnia parasitica*	*S6*	Enzootic	OQ678619
FF.10.02.19	F	20/12/2018	Tank water	*Saprolegnia parasitica*	*S6*	Enzootic	OQ678620
FF.10.09	F	20/12/2018	Skin	*Saprolegnia parasitica*	*S6*	Enzootic	OQ678621
FF.15.08	F	30/03/2019	Fin	*Pythium sp.2*		Enzootic	OQ678622
FF.21.17	F	27/11/2019	Skin	*Saprolegnia parasitica*	*S6*	Enzootic	OQ678623
FF.43	F	05/05/2019	N/a	*Pythium sp.2*		Enzootic	OQ678624
GG.02.01	G	18/08/2017	Skin	*Pythium coloratum*		Enzootic	OQ678625

GG.02.01.12	G	18/08/2017	Tank water	*Pythium coloratum*		Enzootic	OQ678626
GG.02.01.24	G	18/08/2017	Tank water	*Verticillium sp.*		Enzootic	OQ678627
GG.06.18	G	19/09/2017	Fin	*Saprolegnia parasitica*	*S2*	Epizootic	OQ678628
GG.07.02	G	20/09/2017	Fin	*Saprolegnia parasitica*	*S2*	Epizootic	OQ678629
GG.07.02.02	G	20/09/2017	Tank water	*Verticillium sp.*		Epizootic	OQ678630
GG.07.02.20	G	20/09/2017	Tank water	*Pythium pyrilobum*		Epizootic	OQ678631
GG.07.13	G	20/09/2017	Skin	*Saprolegnia parasitica*	*S2*	Epizootic	OQ678632
GG.11.02.13	G	31/10/2017	Tank water	*Mortierella turficola*		Enzootic	OQ678633
GG.11.08.A	G	31/10/2017	Tank water	*Mortierella turficola*		Enzootic	OQ678634
GG.11.14.A	G	31/10/2017	Tank water	*Mortierella turficola*		Enzootic	OQ678635
GG.11.16	G	31/10/2017	Fin	*Pythium oopapillum*		Enzootic	OQ678636
GG.13.10	G	13/11/2017	Fin	*Saprolegnia diclina*		Enzootic	OQ678637
GG.14.02.15	G	14/11/2017	Tank water	*Saprolegnia diclina*		Enzootic	OQ678638
GG.25.07	G	08/02/2018	Skin	*Mortierella sp.1*		Enzootic	OQ678639
GG.25.09	G	08/02/2018	Skin	*Mortierella sp.1*		Enzootic	OQ678640
GG.33.02.10	G	09/04/2018	Tank water	*Saprolegnia parasitica*	*S2*	Epizootic	OQ678641
GG.33.02.14	G	09/04/2018	Tank water	*Saprolegnia parasitica*	*S2*	Epizootic	OQ678642
GG.33.02.21	G	09/04/2018	Tank water	*Saprolegnia parasitica*	*S2*	Epizootic	OQ678643
GG.35.02.12	G	20/04/2018	Tank water	*Saprolegnia parasitica*	*S2*	Epizootic	OQ678644
GG.35.05	G	20/04/2018	Skin	*Saprolegnia parasitica*	*S2*	Epizootic	OQ678645
GG.35.15	G	20/04/2018	Skin	*Saprolegnia parasitica*	*S2*	Epizootic	OQ678646
GG.37.02.20	G	01/06/2018	Tank water	*Saprolegnia diclina*		Enzootic	OQ678647
GG.37.10	G	01/06/2018	Fin	*Leptolegnia sp.*		Enzootic	OQ678648
GG.42.01	G	03/07/2018	Skin	*Bjerkandera adusta*		Enzootic	OQ678649
GG.42.08	G	03/07/2018	Fin	*Saprolegnia parasitica*	*S2*	Enzootic	OQ678650

GG.45.02.12	G	06/08/2018	Tank water	*Pythium coloratum*		Enzootic	OQ678651
GG.45.10	G	06/08/2018	Fin	*Saprolegnia parasitica*	*S2*	Enzootic	OQ678652
GG.48.02	G	24/08/2018	Fin	*Saprolegnia parasitica*	*S5*	Epizootic	OQ678653
GG.48.02.20	G	24/08/2018	Tank water	*Pythium sp.3*		Epizootic	OQ678654
GG.48.04	G	24/08/2018	Fin	*Saprolegnia parasitica*	*S5*	Epizootic	OQ678655
GG.48.11	G	24/08/2018	Skin	*Saprolegnia parasitica*	*S5*	Epizootic	OQ678656
GG.48.12	G	24/08/2018	Fin	*Saprolegnia parasitica*	*S2*	Epizootic	OQ678657
GG.48.16	G	24/08/2018	Fin	*Saprolegnia parasitica*	*S3*	Epizootic	OQ678658
GG.55.02.01	G	08/10/2018	Tank water	*Pythium undulatum*		Enzootic	OQ678659
GG.55.02.09	G	08/10/2018	Tank water	*Pythium undulatum*		Enzootic	OQ678660
GG.56.02.04	G	15/10/2018	Tank water	*Pythium paddicum*		Enzootic	OQ678661
GG.56.10	G	15/10/2018	Fin	*Saprolegnia parasitica*	*S2*	Enzootic	OQ678662
GG.61.02	G	23/11/2018	Fin	*Saprolegnia parasitica*	*S2*	Enzootic	OQ678663
GG.65.02.05	G	20/12/2018	Tank water	*Mortierella sp.5*		Enzootic	OQ678664
GG.87.08	G	26/07/2019	Fin	*Saprolegnia parasitica*	*S2*	Enzootic	OQ678665
GG.93.02.18	G	30/08/2019	Tank water	*Pythium coloratum*		Enzootic	OQ678666
HH.02.02.11	H	18/08/2017	Tank water	*Pythium pyrilobum*		Enzootic	OQ678667
HH.02.02.13	H	18/08/2017	Tank water	*Pythium pyrilobum*		Enzootic	OQ678668
HH.02.02.15	H	18/08/2017	Tank water	*Pythium anandrum*		Enzootic	OQ678669
HH.02.02.15	H	18/08/2017	Tank water	*Pythium pyrilobum*		Enzootic	OQ678670
HH.02.02.19	H	18/08/2017	Tank water	*Pythium anandrum*		Enzootic	OQ678671
HH.02.02.19	H	18/08/2017	Tank water	*Pythium pyrilobum*		Enzootic	OQ678672
HH.08.02.05	H	28/09/2017	Tank water	*Pythium pyrilobum*		Enzootic	OQ678673
HH.08.02.06	H	28/09/2017	Tank water	*Pythium anandrum*		Enzootic	OQ678674
HH.17.02.11	H	04/04/2018	Tank water	*Pythium sp.5*		Epizootic	OQ678675

HH.20.01	H	25/04/2018	Skin	*Mortierella sp.3*		Epizootic	OQ678676
HH.20.02.09	H	25/04/2018	Tank water	*Pythium pyrilobum*		Epizootic	OQ678677
HH.20.02.11	H	25/04/2018	Tank water	*Pythium coloratum*		Epizootic	OQ678678
HH.20.03	H	25/04/2018	Skin	*Mortierella sp.3*		Epizootic	OQ678679
HH.20.04	H	25/04/2018	Fin	*Mortierella sp.3*		Epizootic	OQ678680
HH.20.11	H	25/04/2018	Skin	*Saprolegnia parasitica*	*S2*	Epizootic	OQ678681
HH.20.12	H	25/04/2018	Fin	*Saprolegnia parasitica*	*S2*	Epizootic	OQ678682
HH.25.09	H	30/05/2018	Skin	*Mortierella sp.1*		Enzootic	OQ678683
HH.26.02.11	H	13/06/2018	Tank water	*Pythium coloratum*		Enzootic	OQ678684
HH.27.02.02	H	22/06/2018	Tank water	*Pythium coloratum*		Enzootic	OQ678685
HH.27.14	H	22/06/2018	Fin	*Pythium undulatum*		Enzootic	OQ678686
HH.27.18	H	22/06/2018	Fin	*Mortierella sp.1*		Enzootic	OQ678687
HH.30.02.16	H	23/07/2018	Tank water	*Pythium sp.4*		Enzootic	OQ678688
HH.31.02.06	H	06/08/2018	Tank water	*Pythium coloratum*		Enzootic	OQ678689
HH.40.02.14	H	15/11/2018	Tank water	*Pythium sp.5*		Enzootic	OQ678690
HH.41.02.17	H	23/11/2018	Tank water	*Phytophthora gonapodyides*		Enzootic	OQ678691
HH.43.02.11	H	14/12/2018	Tank water	*Pythium sp.5*		Enzootic	OQ678692
HH.43.18	H	14/12/2018	Fin	*Mortierella sp.8*		Enzootic	OQ678693
HH.50.02.23	H	07/02/2019	Tank water	*Mortierella turficola*		Enzootic	OQ678694
HH.59.09	H	09/04/2019	Skin	*Saprolegnia parasitica*	*S2*	Epizootic	OQ678695
HH.59.16	H	09/04/2019	Fin	*Saprolegnia diclina*		Epizootic	OQ678696
HH.74.02.07	H	30/07/2019	Tank water	*Saprolegnia parasitica*	*S2*	Enzootic	OQ678697
HH.74.02.11	H	30/07/2019	Tank water	*Pythium sp.7*		Enzootic	OQ678698
HH.74.02.15	H	30/07/2019	Tank water	*Unknown Fungi*		Enzootic	OQ678699
HH.78.02.18	H	04/09/2019	Tank water	*Pythium undulatum*		Epizootic	OQ678700

HH.78.04	H	04/09/2019	Fin	*Saprolegnia parasitica*	*S2*	Epizootic	OQ678701
HH.80.01	H	17/09/2019	Skin	*Saprolegnia diclina*		Epizootic	OQ678702
HH.82.10	H	03/11/2019	Fin	*Saprolegnia parasitica*	*S2*	Enzootic	OQ678703
HH.82.16	H	03/11/2019	Fin	*Saprolegnia parasitica*	*S2*	Enzootic	OQ678704
II.01.01.09	I	16/08/2017	Tank water	*Pythium coloratum*		Enzootic	OQ678705
II.02.01	I	16/08/2017	Skin	*Saprolegnia parasitica*	*S2*	Enzootic	OQ678706
II.02.02	I	16/08/2017	Fin	*Saprolegnia parasitica*	*S2*	Enzootic	OQ678707
II.02.02.24	I	16/08/2017	Tank water	*Saprolegnia delica*		Enzootic	OQ678708
II.02.04	I	16/08/2017	Fin	*Saprolegnia parasitica*	*S2*	Enzootic	OQ678709
II.02.06	I	16/08/2017	Fin	*Saprolegnia parasitica*	*S2*	Enzootic	OQ678710
II.02.08	I	16/08/2017	Fin	*Saprolegnia parasitica*	*S2*	Enzootic	OQ678711
II.02.10	I	16/08/2017	Fin	*Saprolegnia parasitica*	*S2*	Enzootic	OQ678712
II.02.12	I	16/08/2017	Fin	*Saprolegnia parasitica*	*S2*	Enzootic	OQ678713
II.11.02.22	I	18/10/2017	Tank water	*Saprolegnia diclina*		Enzootic	OQ678714
II.11.11	I	18/10/2017	Skin	*Microdochium sp.*		Enzootic	OQ678715
II.13.08	I	02/11/2017	Fin	*Mortierella sp.2*		Enzootic	OQ678716
II.13.18	I	02/11/2017	Fin	*Mortierella sp.2*		Enzootic	OQ678717
II.15.02.03	I	16/11/2017	Tank water	*Saprolegnia diclina*		Enzootic	OQ678718
II.23.02.05	I	10/01/2018	Tank water	*Pythium coloratum*		Enzootic	OQ678719
II.26.09	I	12/02/2018	Skin	*Mortierella sp.5*		Enzootic	OQ678720
II.26.13	I	12/02/2018	Skin	*Mortierella sp.6*		Enzootic	OQ678721
II.35.02.16	I	03/05/2018	Tank water	*Achlya sp.1*		Epizootic	OQ678722
II.35.03	I	03/05/2018	Skin	*Saprolegnia parasitica*	*S2*	Epizootic	OQ678723
II.36.02.21	I	11/05/2018	Tank water	*Pythium undulatum*		Enzootic	OQ678724
II.47.06	I	30/07/2018	Fin	*Saprolegnia parasitica*	*S2*	Enzootic	OQ678725

II.47.08	I	30/07/2018	Fin	*Saprolegnia parasitica*	*S2*	Enzootic	OQ678726
II.55.05	I	28/09/2018	Skin	*Saprolegnia parasitica*	*S2*	Epizootic	OQ678727
II.55.07	I	28/09/2018	Skin	*Saprolegnia parasitica*	*S2*	Epizootic	OQ678728
II.55.15	I	28/09/2018	Skin	*Saprolegnia parasitica*	*S2*	Epizootic	OQ678729
II.56.04	I	11/10/2018	Fin	*Mortierella hyalina*		Epizootic	OQ678730
II.56.06	I	11/10/2018	Fin	*MortierelJJ. sp.10*		Epizootic	OQ678731
II.56.10	I	11/10/2018	Fin	*Mortierella hyalina*		Epizootic	OQ678732
II.56.14	I	11/10/2018	Fin	*Mortierella hyalina*		Epizootic	OQ678733
II.61.02.17	I	13/11/2018	Tank water	*Pythium flevoense*		Enzootic	OQ678734
II.61.16	I	13/11/2018	Fin	*Saprolegnia diclina*		Enzootic	OQ678735
II.70.02.22	I	15/01/2019	Tank water	*Saprolegnia diclina*		Epizootic	OQ678736
II.73.12	I	22/02/2019	Fin	*Mortierella sp.9*		Epizootic	OQ678737
II.93.09	I	18/07/2019	Skin	*Mortierella sp.11*		Epizootic	OQ678738
JJ.01.02.05	J	16/11/2017	Tank water	*Saprolegnia australis*		Epizootic	OQ678739
JJ.01.02.16	J	16/11/2017	Tank water	*Saprolegnia australis*		Epizootic	OQ678740
JJ.01.06	J	16/11/2017	Fin	*Saprolegnia australis*		Epizootic	OQ678741
JJ.01.08	J	16/11/2017	Fin	*Cephalodsporium sp.*		Epizootic	OQ678742
JJ.01.18-A	J	16/11/2017	Fin	*Saprolegnia australis*		Epizootic	OQ678743
JJ.01.18-B	J	16/11/2017	Tank water	*Mortierella sp.4*		Epizootic	OQ678744
JJ.06.02.01	J	11/04/2018	Tank water	*Saprolegnia australis*		Enzootic	OQ678745
JJ.06.02.12	J	11/04/2018	Tank water	*Saprolegnia australis*		Enzootic	OQ678746
JJ.14.02.09	J	01/06/2018	Tank water	*Saprolegnia australis*		Enzootic	OQ678747
JJ.14.02.11	J	01/06/2018	Tank water	*Saprolegnia australis*		Enzootic	OQ678748
JJ.14.02.16	J	01/06/2018	Tank water	*Saprolegnia australis*		Enzootic	OQ678749
JJ.14.08	J	01/06/2018	Fin	*Achlya sp.4*		Enzootic	OQ678750

JJ.14.18	J	01/06/2018	Fin	*Saprolegnia australis*		Enzootic	OQ678751
JJ.26.08	J	22/08/2018	Fin	*Saprolegnia delica*		Enzootic	OQ678752
JJ.34.02	J	18/10/2018	Fin	*Mortierella hyalina*		Enzootic	OQ678753
JJ.34.04	J	18/10/2018	Fin	*Mortierella hyalina*		Enzootic	OQ678754
JJ.34.08	J	18/10/2018	Fin	*Mortierella hyalina*		Enzootic	OQ678755
JJ.34.12	J	18/10/2018	Fin	*Mortierella hyalina*		Enzootic	OQ678756
JJ.34.14	J	18/10/2018	Fin	*Saprolegnia australis*		Enzootic	OQ678757
JJ.34.16-A	J	18/10/2018	Fin	*Mortierella hyalina*		Enzootic	OQ678758
JJ.34.16-B	J	18/10/2018	Fin	*Pythium flevoense*		Enzootic	OQ678759
JJ.40.12	J	26/11/2018	Fin	*Saprolegnia australis*		Enzootic	OQ678760
JJ.49.02	J	29/01/2019	Fin	*Mortierella hyalina*		Epizootic	OQ678761
JJ.49.03	J	29/01/2019	Skin	*Saprolegnia delica*		Epizootic	OQ678762
JJ.49.08	J	29/01/2019	Fin	*Saprolegnia delica*		Epizootic	OQ678763
JJ.49.10	J	29/01/2019	Fin	*Saprolegnia delica*		Epizootic	OQ678764
JJ.52.18	J	19/02/2019	Fin	*Saprolegnia diclina*		Epizootic	OQ678765
JJ.53.02.03	J	26/02/2019	Tank water	*Saprolegnia delica*		Epizootic	OQ678766
JJ.53.02.04	J	26/02/2019	Tank water	*Saprolegnia delica*		Epizootic	OQ678767
JJ.54.02.01	J	06/03/2019	Tank water	*Saprolegnia parasitica*	*S2*	Enzootic	OQ678768
JJ.62.02.06	J	17/05/2019	Tank water	*Saprolegnia delica*		Enzootic	OQ678769
JJ.62.02.20	J	17/05/2019	Tank water	*Achlya sp.2*		Enzootic	OQ678770
JJ.66.02.21	J	10/06/2019	Tank water	*Pythium flevoense*		Enzootic	OQ678771
KK.01.07	K	30/10/2017	Skin	*Mortierella sp.5*		Enzootic	OQ678772
KK.01.18	K	30/10/2017	Fin	*Mortierella sp.6*		Enzootic	OQ678773
KK.02.12	K	30/11/2017	Fin	*Mortierella sp.1*		Enzootic	OQ678774
KK.04.02.05	K	24/01/2018	Tank water	*Phytophthora chlamydospora*		Enzootic	OQ678775

KK.04.10	K	24/01/2018	Fin	*Mortierella hyalina*		Enzootic	OQ678776
KK.10.01	K	08/11/2018	Skin	*Saprolegnia parasitica*	*S2*	Enzootic	OQ678777
LL.01.02.24	L	18/10/2017	Tank water	*Pythium sp.5*		Enzootic	OQ678790
LL.01.07	L	18/10/2017	Skin	*Saprolegnia parasitica*	*S2*	Enzootic	OQ678791
LL.01.09	L	18/10/2017	Skin	*Saprolegnia parasitica*	*S2*	Enzootic	OQ678792
LL.02.02	L	13/11/2017	Fin	*Saprolegnia parasitica*	*S2*	Enzootic	OQ678793
LL.02.02.16	L	13/11/2017	Tank water	*Saprolegnia diclina*		Enzootic	OQ678794
LL.02.09	L	13/11/2017	Skin	*Saprolegnia parasitica*	*S2*	Enzootic	OQ678795
LL.04.14	L	14/01/2018	Fin	*Saprolegnia parasitica*	*S2*	Enzootic	OQ678796
LL.04.16	L	14/01/2018	Fin	*Saprolegnia parasitica*	*S2*	Enzootic	OQ678797
LL.04.18	L	14/01/2018	Fin	*Saprolegnia parasitica*	*S2*	Enzootic	OQ678798
LL.05.06	L	16/01/2018	Fin	*Saprolegnia parasitica*	*S2*	Enzootic	OQ678799
LL.05.10	L	16/01/2018	Fin	*Saprolegnia parasitica*	*S2*	Enzootic	OQ678800
MM.01.13	M	30/10/2017	Skin	*Deconica citrispora*		Enzootic	OQ678801
MM.03.02.06	M	20/12/2017	Tank water	*Pythium coloratum*		Enzootic	OQ678802
MM.13.02.23	M	30/08/2018	Tank water	*Pythium sp.2*		Enzootic	OQ678803
MM.13.10	M	30/08/2018	Fin	*Pythium sp.8*		Enzootic	OQ678804
MM.13.14	M	30/08/2018	Fin	*Achlya sp.5*		Enzootic	OQ678805
MM.13.16	M	30/08/2018	Fin	*Saprolegnia parasitica*	*S2*	Enzootic	OQ678806
MM.14.06	M	07/09/2018	Fin	*Saprolegnia parasitica*	*S2*	Epizootic	OQ678807
MM.14.12	M	07/09/2018	Fin	*Saprolegnia parasitica*	*S2*	Epizootic	OQ678808
MM.14.14	M	07/09/2018	Fin	*Saprolegnia delica*		Epizootic	OQ678809
MM.15.02.20	M	03/10/2018	Tank water	*Saprolegnia ferax*		Enzootic	OQ678810
MM.15.10	M	03/10/2018	Fin	*Mortierella hyalina*		Enzootic	OQ678811
MM.16.02	M	08/11/2018	Fin	*Saprolegnia parasitica*	*S2*	Enzootic	OQ678812

MM.16.05	M	08/11/2018	Skin	*Saprolegnia parasitica*	*S2*	Enzootic	OQ678813
MM.16.07	M	08/11/2018	Skin	*Saprolegnia parasitica*	*S2*	Enzootic	OQ678814
MM.16.08	M	08/11/2018	Fin	*Saprolegnia parasitica*	*S2*	Enzootic	OQ678815
MM.16.09	M	08/11/2018	Skin	*Saprolegnia parasitica*	*S2*	Enzootic	OQ678816
MM.16.12	M	08/11/2018	Fin	*Saprolegnia parasitica*	*S2*	Enzootic	OQ678817
MM.16.18	M	08/11/2018	Fin	*Saprolegnia parasitica*	*S2*	Enzootic	OQ678818
MM.26.02	M	N/a	Fin	*Saprolegnia parasitica*	*S2*	Enzootic	OQ678819
NN.25.03	N	24/07/2019	Skin	*Plebia rufa*		Enzootic	OQ678778
NN.26.02.02	N	14/01/2020	Tank water	*Saprolegnia australis*		Enzootic	OQ678779
NN.26.02.09	N	14/01/2020	Tank water	*Saprolegnia parasitica*	*S2*	Enzootic	OQ678780
NN.26.03	N	14/01/2020	Skin	*Saprolegnia parasitica*	*S2*	Enzootic	OQ678781
NN.26.04	N	14/01/2020	Fin	*Saprolegnia parasitica*	*S2*	Enzootic	OQ678782
NN.26.07	N	14/01/2020	Skin	*Saprolegnia parasitica*	*S2*	Enzootic	OQ678783
NN.26.09	N	14/01/2020	Skin	*Saprolegnia parasitica*	*S2*	Enzootic	OQ678784
NN.26.10	N	14/01/2020	Fin	*Saprolegnia parasitica*	*S2*	Enzootic	OQ678785
NN.26.14	N	14/01/2020	Fin	*Saprolegnia australis*		Enzootic	OQ678786
NN.26.15	N	14/01/2020	Skin	*Pythium coloratum*		Enzootic	OQ678787
NN.26.16	N	14/01/2020	Fin	*Saprolegnia parasitica*	*S2*	Enzootic	OQ678788
NN.26.18	N	14/01/2020	Fin	*Saprolegnia parasitica*	*S2*	Enzootic	OQ678789

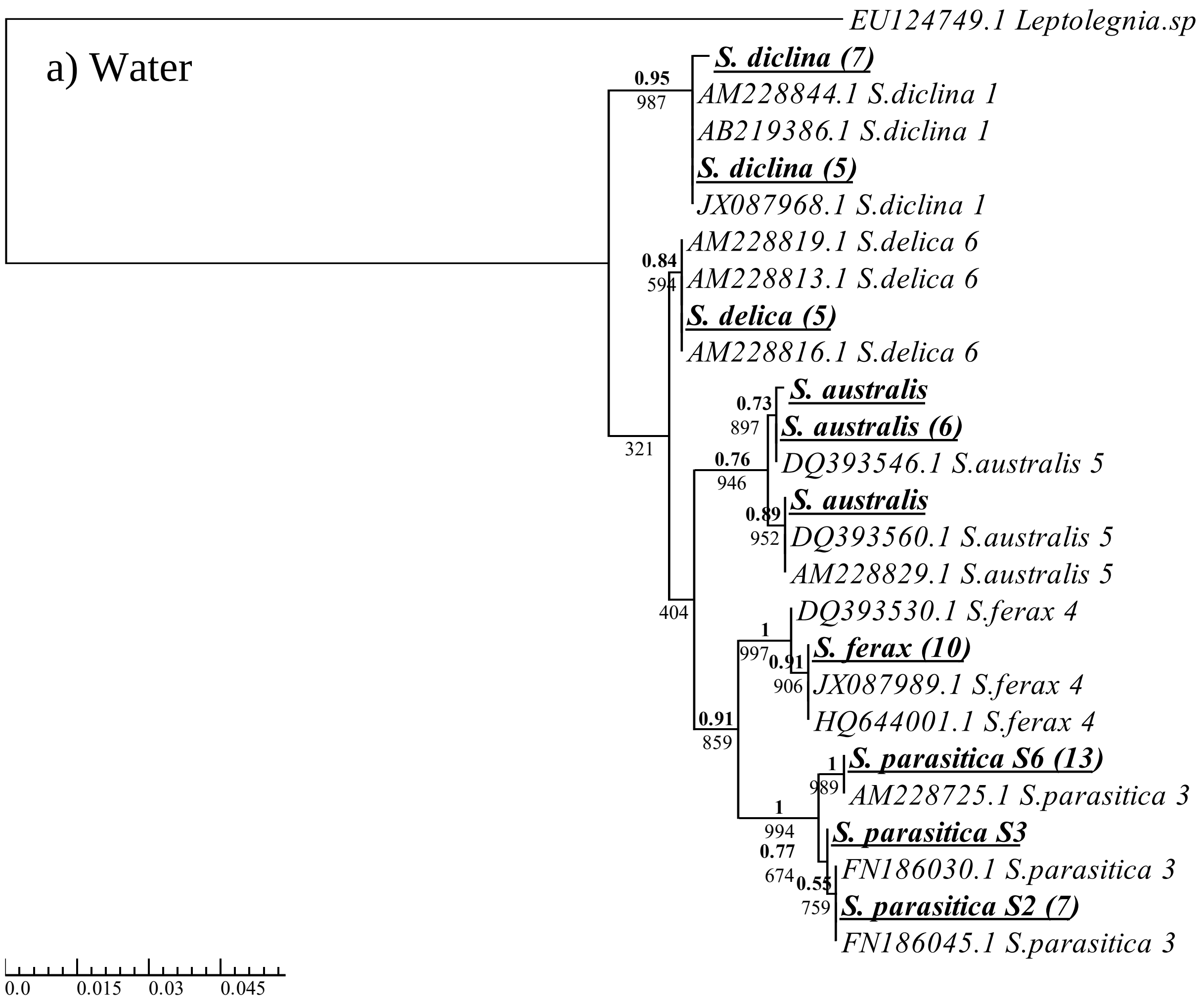
EU124749.1 Leptolegnia.sp
a) Water
S. diclina (7)
AM228844.1 S.diclina 1
AB219386.1 S.diclina 1
S. diclina (5)
JX087968.1 S.diclina 1
AM228819.1 S.delica 6
AM228813.1 S.delica 6
S. delica (5)
AM228816.1 S.delica 6
S. australis
S. australis (6)
DQ393546.1 S.australis 5
S. australis
DQ393560.1 S.australis 5
AM228829.1 S.australis 5
DQ393530.1 S.ferax 4
S. ferax (10)
JX087989.1 S.ferax 4
HQ644001.1 S.ferax 4
S. parasitica S6 (13)
AM228725.1 S.parasitica 3
S. parasitica S3
FN186030.1 S.parasitica 3
S. parasitica S2 (7)
FN186045.1 S.parasitica 3
0.95
987
0.84
594
321
0.73
897
0.76
946
0.89
952
404
1
997
0.91
906
0.91
859
1
989
1
994
0.77
674
0.55
759
0.0
0.015
0.03
0.045

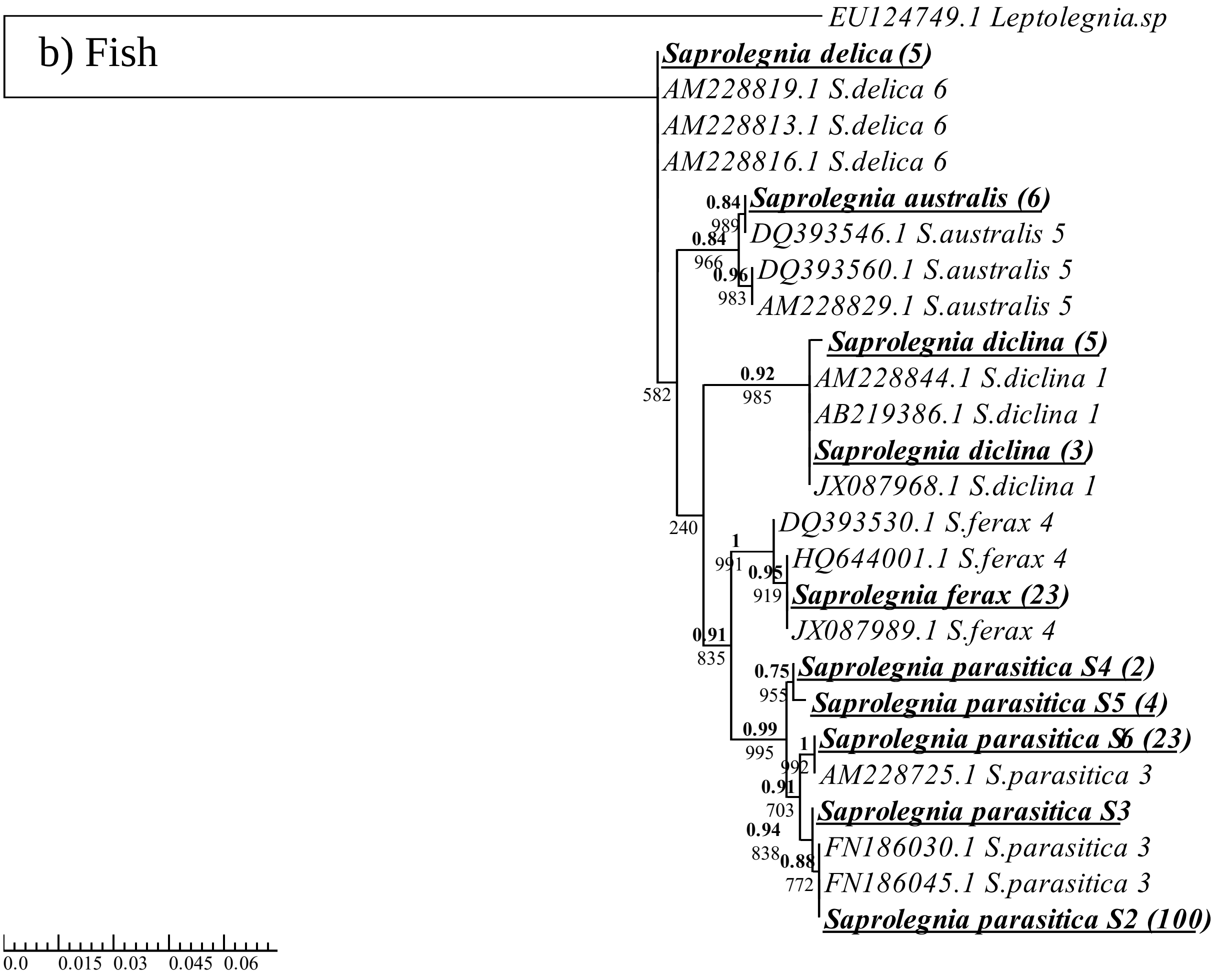

Figure S1. Phylogenetic summary of all *Saprolegnia* species divided between water and fish samples. Isolates sampled are divided between those sampled from a) Water and b) Fish. A single consensus sequence was used for isolates with identical ITS sequences, with the number of isolates for each consensus in brackets next to the species name. Isolates from this study are boldened and underlined. Reference sequences were taken from [14] and are listed with their NCBI accession number, species names, and cluster number as designated by the reference study. Phy Maximum Likelihood tree and Bayesian inference for both trees were produced using the HKY+G model. Supporting values for each branch are displayed with Maximum-Likelihood bootstrap values below and Bayesian support values above (bold).

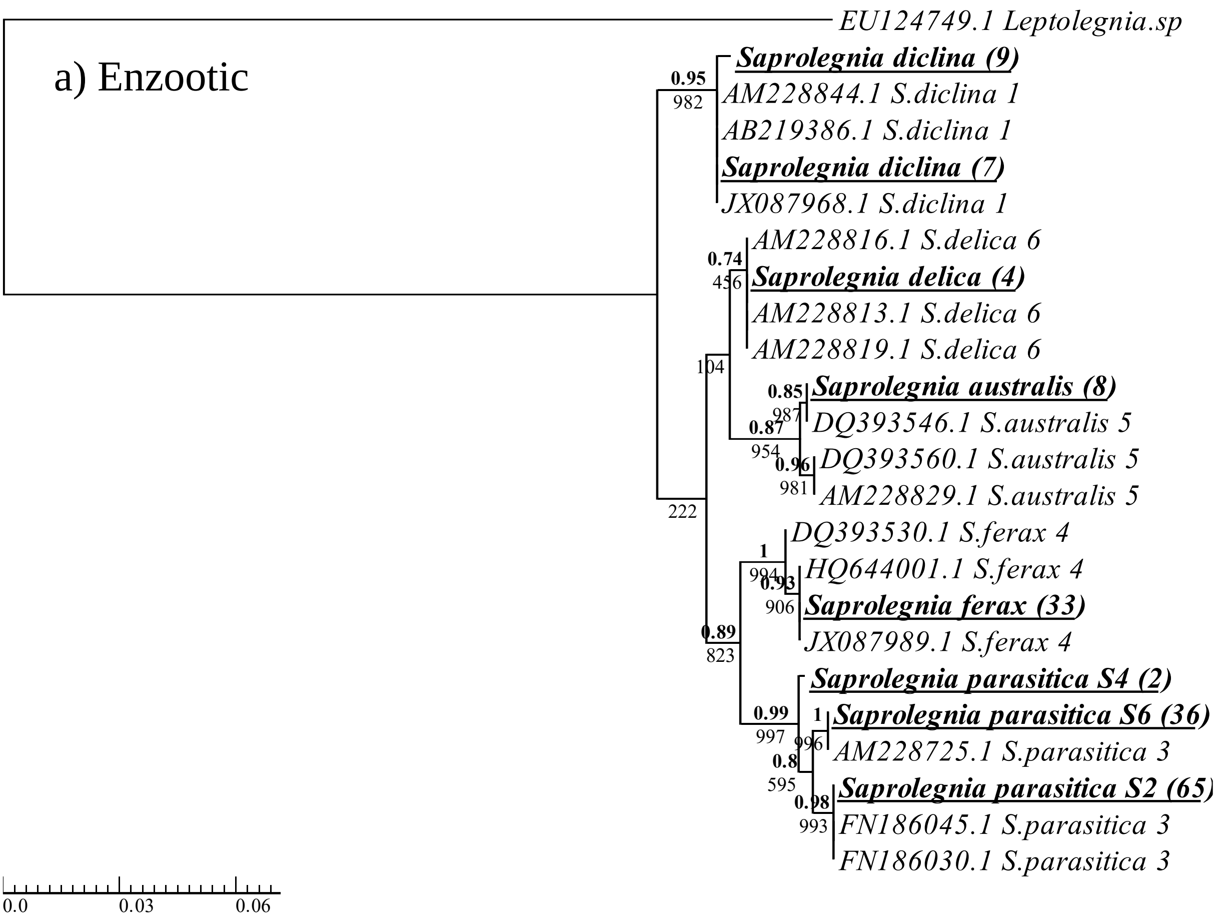

a) Enzootic
EU124749.1 Leptolegnia.sp
Saprolegnia diclina (9)
AM228844.1 S.diclina 1
AB219386.1 S.diclina 1
Saprolegnia diclina (7)
JX087968.1 S.diclina 1
AM228816.1 S.delica 6
Saprolegnia delica (4)
AM228813.1 S.delica 6
AM228819.1 S.delica 6
Saprolegnia australis (8)
DQ393546.1 S.australis 5
DQ393560.1 S.australis 5
AM228829.1 S.australis 5
DQ393530.1 S.ferax 4
HQ644001.1 S.ferax 4
Saprolegnia ferax (33)
JX087989.1 S.ferax 4
Saprolegnia parasitica S4 (2)
Saprolegnia parasitica S6 (36)
AM228725.1 S.parasitica 3
Saprolegnia parasitica S2 (65)
FN186045.1 S.parasitica 3
FN186030.1 S.parasitica 3
0.95
982
0.74
456
104
0.85
987
0.87
954
0.96
981
222
1
994
0.93
906
0.89
823
0.99
997
1
996
0.8
595
0.98
993
0.0
0.03
0.06

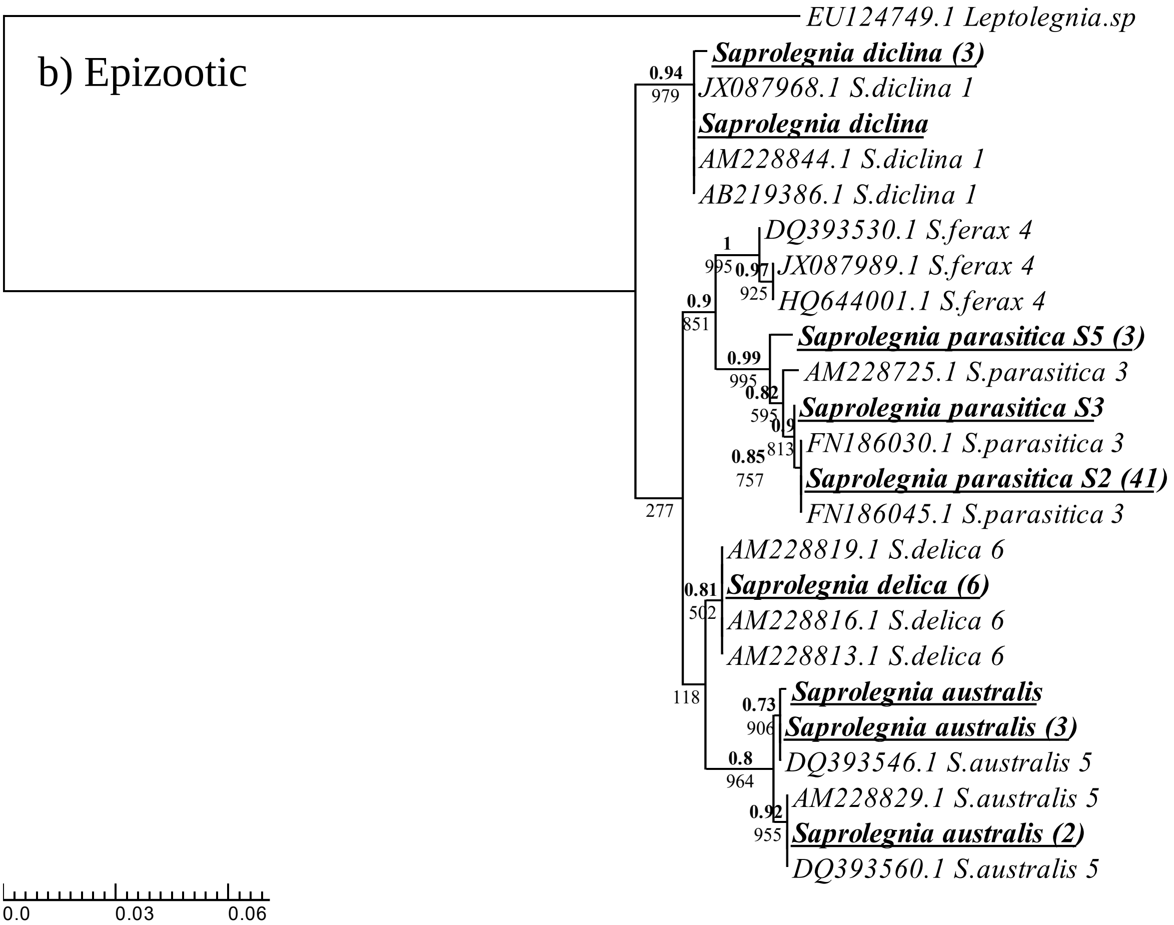

Figure S2. Phylogenetic summary of all *Saprolegnia* species divided between enzootic and epizootic sampling periods. Isolates sampled are divided between those sampled from a) Enzootic periods, and b) Epizootic periods. A single consensus sequence was used for isolates with identical ITS sequences, with the number of isolates for each consensus in brackets next to the species name. Isolates from this study are boldened and underlined. Reference sequences were taken from [14] and are listed with their NCBI accession number, species names, and cluster number as designated by the reference study. Phy Maximum-likelihood tree and Bayesian inference for both trees were produced using the HKY+G model. Supporting values for each branch are displayed with Maximum-Likelihood bootstrap values below and Bayesian support values above (bold).
